# An Epileptic EEG Detection Method Based on Data Augmentation and Lightweight Neural Network

**DOI:** 10.1109/JTEHM.2023.3308196

**Published:** 2023-08-24

**Authors:** Chenlong Wang, Lei Liu, Wenhai Zhuo, Yun Xie

**Affiliations:** School of AutomationGuangdong University of Technology 47870 Guangzhou 523083 China

**Keywords:** Electroencephalography, epilepsy detection, deep learning, data augmentation, lightweight neural networks.

## Abstract

Objective: Epilepsy, an enduring neurological disorder, afflicts approximately 65 million individuals globally, significantly impacting their physical and mental wellbeing. Traditional epilepsy detection methods are labor-intensive, leading to inefficiencies. Although deep learning techniques for brain signal detection have gained traction in recent years, their clinical application advancement is hindered by the significant requirement for high-quality data and computational resources during training. Methods & Results: The neural network training initially involved merging two datasets of different data quality, namely Bonn University datasets and CHB-MIT datasets, to bolster its generalization capabilities. To tackle the issues of dataset size and class imbalance, we employed small window segmentation and Synthetic Minority Over-sampling Technique (SMOTE). algorithms to augment and equalize the data. A streamlined neural network architecture was then proposed, drastically reducing the model’s training parameters. Notably, a model trained with a mere 9,371 parameters yielded impressive results. The three-classification task on the combined dataset delivered an accuracy of 98.52%, sensitivity of 97.99%, specificity of 99.35%, and precision of 98.44%.Conclusion: The experimental findings of this study underscore the superiority of the proposed method over existing approaches in both model size reduction and accuracy enhancement. As a result, it is more apt for deployment in low-cost, low computational hardware devices, including wearable technology, and various clinical applications. Clinical and Translational Impact Statement— This study is a Pre-Clinical Research. The lightweight neural network is easily deployed on hardware device for real-time epileptic EEG detection.

## I. Introduction

Epilepsy, a chronic ailment, ranks as the second most prevalent neurological disorder [1], [2]. It manifests as transient brain dysfunction stemming from sporadic, abnormal neuronal firing.

In order to effectively detect and recognize the epilepsy, one effective way to determine this is through the electroencephalogram (EEG). The EEG is a weak electrical signal produced by the interaction of neural activity within the brain [3]. Through EEG testing, clinical expert can diagnose patients and help select appropriate treatment options.

In the clinical setting, clinical experts make diagnoses by examining the recorded EEG data and make comprehensive judgments based on the patient’s clinical symptoms. Given the irregular and transient nature of seizures [4], long-term EEG recordings are necessary. This requires experts to recognize seizure characteristics from a large amount of recorded EEG data over a long period of time. This time-consuming and heavily empirical approach [5] can lead to expert fatigue and possible misdiagnosis due to prolonged diagnosis.

The recent surge in automated computer-aided epilepsy detection research bifurcates into two domains: machine learning and deep learning.

Machine learning processes EEG data through four stages: noise reduction, feature extraction, feature selection, and classification. The feature extraction stage is critical as it impacts the classifier’s effectiveness. Standard feature extraction methods include time-frequency analysis [6], non-linear dynamics [7], and statistical features [8]. For instance, the method delineated in [6] employs the Discrete Wavelet Transform (DWT) for noise reduction, followed by frequency domain wavelet decomposition for feature extraction, culminating in Nonlinear Vector Decomposition Neural Network (NVDN) classification. This method boasts a 95.60% accuracy on the Bonn University dataset. However, these machine learning techniques, while effective on specific datasets, falter on others. Their reliance on manual feature design both burdens the process and curtails generalizability.

Deep learning based detection methods can automatically extract high-level features from data through powerful computational capabilities. These features are less interpretable but possess desirable classification results. Key models in deep learning, such as Convolutional Neural Networks (CNNs) and Recurrent Neural Networks (RNNs), can directly learn feature representations for classification, bypassing the need for data pre-processing. Compared to manually designed feature extraction methods, deep learning algorithms demonstrate more robust feature learning and superior detection performance [9], [10]. For example, in [11], the authors used the STFT algorithm to convert EEG signals to 2D image data, and then used some pre-trained model frameworks (VGG, AlexNet and EfficiectNet) in the field of computer vision to recognize the image data. The EfficientNet-B7 model, despite its 66 million parameters [12], only achieved a 93.00% accuracy on the Bonn University dataset. Such models, while computationally intensive, often underperform due to limited EEG sample availability.

**FIGURE 1.**
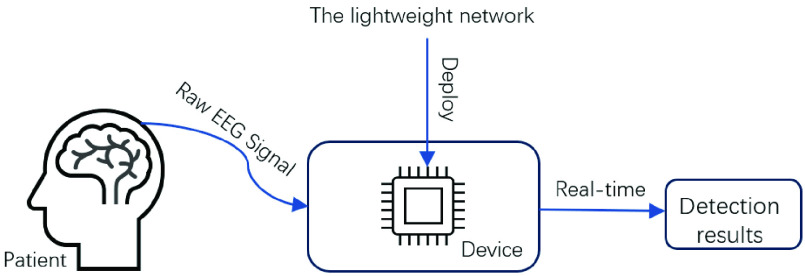
The method of application of this work.

Traditional visual diagnosis by clinical experts is time-consuming, labor-intensive, and inefficient. Machine learning methods heavily depend on hand-extracted features and exhibit poor generalization ability. Deep learning methods can address these issues effectively, but their application in a wide range of clinical applications is limited by their own computational resources and the need for high-quality data. To overcome these challenges, this study proposes a lightweight PCNN-Bi-LSTM hybrid network model. By adjusting the position and number of convolutional kernels of the PCNN, the network’s parameters are reduced, followed by the construction of a Bi-LSTM network, which further extracts the data’s temporal features for classification. This approach achieves high accuracy and a lightweight network architecture, easily deployable on hardware devices for real-time epilepsy EEG detection. Fig. 1 illustrates the proposed method’s schematic figure. Furthermore, this study selects two datasets of different quality for mixed training to increase the dataset’s sample diversity and enhance the trained model’s generalization. We also employ small window segmentation and the SMOTE algorithm to augment the data, achieving dataset expansion and balance, reducing overfitting risk, and further enhancing the model’s generalization ability.

The main contributions of this study are as follows:

• We propose a lightweight neural network architecture for epilepsy detection, called PCNN-BiLSTM, featuring a minimal number of parameters and high recognition accuracy. This method is more suitable for hardware deployment in clinical applications compared to other methods.
• To address the issue of limited EEG data and category imbalance, we utilize a mixture of two datasets of different quality for training, and employ the small window segmentation algorithm and the SMOTE algorithm to expand and balance the data. The network trained from this data exhibits better generalization ability compared to those trained with data used by other methods.

The remainder of this paper is organized as follows: Section II introduces recent related work. Section III describes the methodology proposed in this paper, including the dataset used, the data augmentation algorithm, the neural network architecture, and the model evaluation metrics. Section IV details the experimental setting, including the experimental environment and processing steps. Section V presents the detailed experimental results and analyses. Section VI provides a comparison between the proposed method and other methods, and discusses this paper’s contributions and shortcomings. Finally, Section VII draws conclusions and outlines future directions.

## II. Related Works

Epilepsy detection has made significant strides in recent years, primarily driven by two categories of approaches: machine learning and deep learning. Both approaches have demonstrated remarkable performance in this field.

Machine learning techniques have been employed extensively in epilepsy detection. Bhattacharyya and Pachori [13] utilized Empirical Wavelet Transform (EWT) to extract multiple features from multivariate EEG signals, achieving a classification accuracy of 97.91%. Gupta and Pachori [14] used Fourier-Bessel series expansion (FBSE) to obtain EEG rhythms and extracted features for classification, achieving a triple classification accuracy of 97.3%. Sharma and Pachori [15] proposed a novel Time-frequency representation (TFR) based on the improved eigenvalue decomposition of the Hankel matrix and Hilbert transform (IEVDHM-HT), which reached a classification accuracy of 100%. Sharma et al. [16] used a sparse autoencoder-based deep neural network to extract essential structural details, achieving a classification accuracy of 99.6%. Serna et al. [17] developed an EEG rhythm-specific Taylor-Fourier Filter Bank, achieving a 94.88% accuracy.

Deep learning techniques have also been employed with great success. Raghavendra et al. [18] pioneered the use of CNNs for analyzing epileptic EEG signals, constructing a 13-layer deep CNN model, achieving an accuracy of 88.67%. Vidyaratne et al. [19] developed a Deep Cellular Recurrent Network (DCRNN) framework, achieving an accuracy of 91.3%. Shen et al. [20] utilized image-like data derived from the adjustable Q-wavelet transform, reaching an impressive accuracy of 97.57%. Qiu et al. [21] proposed LightSeizureNet, achieving an accuracy of 97.09%. Tang et al. [22] presented an evolutionary algorithm enhanced model, achieving an accuracy of 98.16%. Varli and Yilmaz [23] created a combined deep learning model, achieving an accuracy of 99.21%. Duan et al. [24] proposed a deep metric learning model, achieving 98.60% accuracy. Salafian et al. [25] developed a Mutual Information-based CNN-Aided Learned factor graphs (MICAL) algorithm, achieving 98.39% accuracy. Qiu et al. [26] proposed a difference attention ResNet-LSTM network (DARLNet), achieving 98.17% accuracy. Zhao et al. [27] used a random channel ordering (OCR) method for data augmentation, and Shyu et al. [28] proposed a model with a combination of inception module and residual module, achieving an accuracy of 97.11%.

With the progressive enhancement of hardware computing power and reduction in costs, the deployment of neural networks has become more accessible. Ai et al. [29] successfully deployed a lightweight convolutional neural network on a TSMC 65nm IP core for EEG-based epilepsy prediction, yielding an impressive accuracy rate of 87.9%. Similarly, Feng et al. [30] deployed an EEGNet on a Xilinx KC705 FPGA for four-class classification utilizing event-related potential (ERP) EEG data, resulting in a remarkable classification accuracy of 96.03%.

A review of the literature reveals that while machine learning depends on the selection of features, most of the feature extraction methods are complex and rely on manual extraction, leading to poor generalization. Deep learning, on the other hand, can automatically extract features from raw data and classify them with excellent recognition results. However, most deep learning models transform one-dimensional digital data into two-dimensional image data or use deeper and more complex network modules, thereby increasing computational complexity. Additionally, most methods do not employ data augmentation algorithms, resulting in trained networks that perform well only on their single dataset but lack overall generalization. Some studies have shown the feasibility of deploying deep learning algorithms on hardware devices, but this may come at the cost of reduced recognition accuracy.

Therefore, this study proposes a method that reduces network complexity and parameters, saving computational resources while ensuring model accuracy. By adopting a hybrid dataset with data augmentation algorithms, the network’s generalization ability is enhanced, facilitating its deployment on wearable hardware devices. This approach has significant clinical implications for enabling affordable and expert-independent epilepsy detection.

## III. Methodology

**FIGURE 2.**
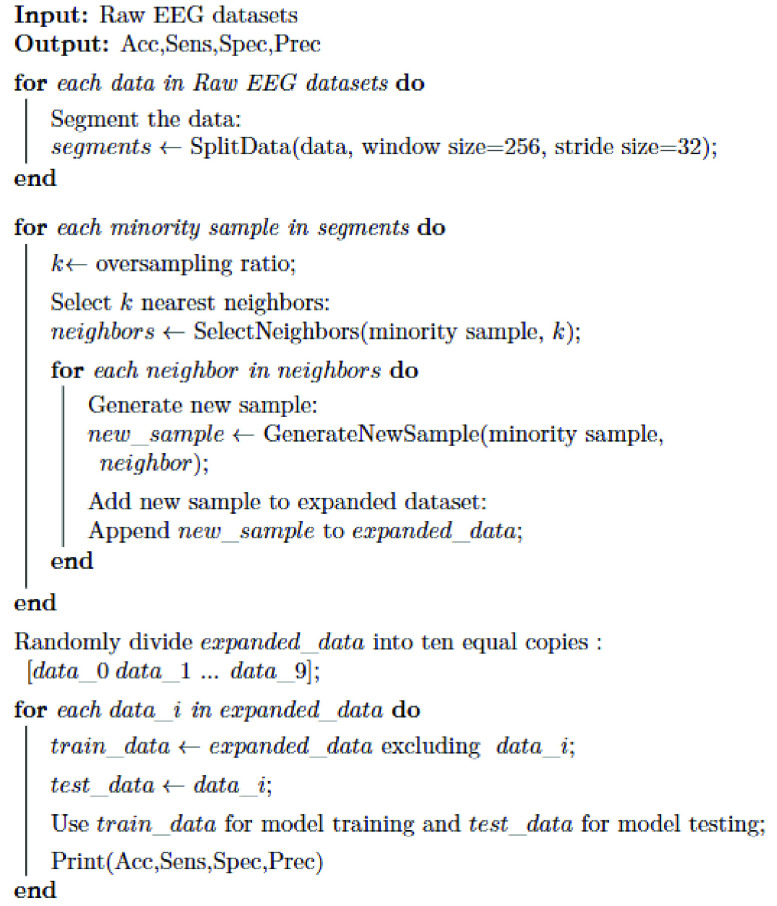
The pseudocode of this work.

The pseudocode of the method is shown in Figure 2.

**FIGURE 3.**
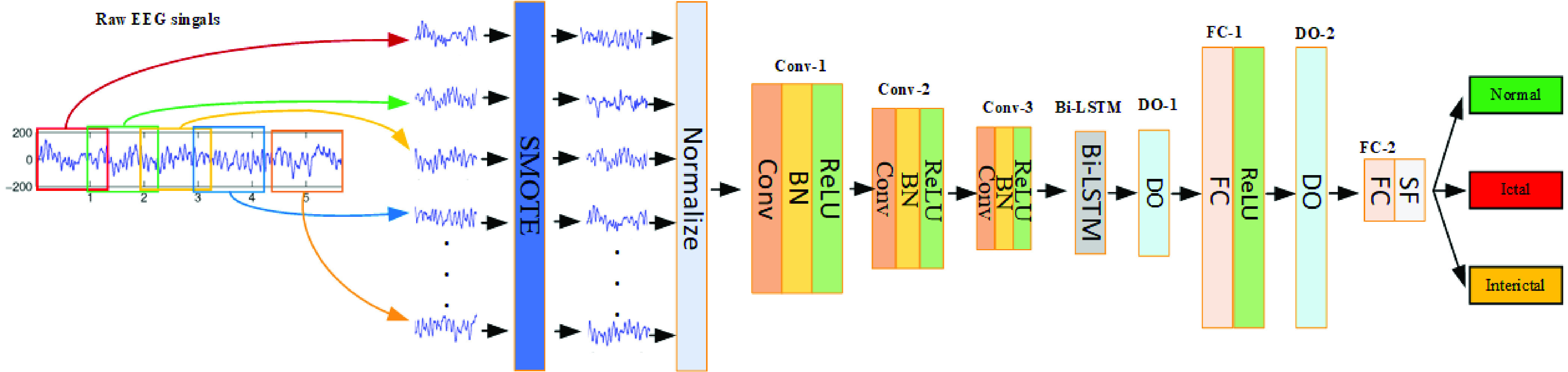
Overall method architecture.

Firstly, the original EEG data from the hybrid dataset is segmented using a sliding window algorithm to transform longer time series data into multiple shorter time series data. This process increases the number of data samples. Secondly, the SMOTE algorithm is used to proportionally expand the smaller sample sizes, balancing the number of samples in each category. Then, the data are randomly divided into ten subsets. Nine of these subsets are used to train our PCNN-BiLSTM hybrid network, which can automatically extract features and perform triple classification. The remaining subset is used for model testing. To reduce randomness, this process is repeated ten times to ensure that each subset is tested. The overall network architecture of our method is depicted in Figure 3.

### A. Dataset

To enhance the generalization capability of deep learning networks across diverse datasets, this study proposes a solution that merges datasets of varying quality for model training. This approach combines datasets from Bonn University and CHB-MIT (specifically, the chb_02) to train our network. By employing this cross-dataset training strategy, we introduce diversity in the data samples, allowing the model to learn more essential features of epilepsy.

#### 1) BONN

The first dataset used in this study is the publicly available epilepsy EEG dataset from Bonn University. This dataset comprises five distinct subsets labeled as A, B, C, D, and E. Each subset contains single-channel EEG data with specific characteristics. Subset A and B consist of scalp EEG data from healthy volunteers, while subsets C and D contain intracranial EEG data from epileptic patients’ non-focal and focal areas, respectively. Subset E contains seizure-related intracranial EEG signals. Each subset comprises 100 files, with each file recording a 23.6-second-long EEG activity at a sampling frequency of 173.61Hz. All data have undergone pre-processing, primarily involving filtering operations and visual detection techniques to eliminate artefactual interference.

#### 2) CHB-MIT

The second dataset used is the publicly accessible pediatric epilepsy EEG dataset provided by CHB-MIT. This dataset includes data obtained from both seizure and interictal periods, adhering to the 10–20 international standard electrode placement system. The dataset comprises multi-channel EEG recordings, with a fixed sampling frequency of 256Hz. It includes a total of 23 recordings derived from 22 subjects.

The Bonn University dataset is divided into three categories: normal, interictal, and ictal. The latter two categories consist of intracranial EEG signals that have undergone rigorous filtering processes. In contrast, the CHB-MIT dataset contains two categories: interictal and ictal, consisting of scalp EEG signals with minimal preprocessing. For consistency, we performed channel averaging on the multi-channel data from the CHB-MIT dataset, resulting in a single-channel signal. The data were then downsampled to 173.61Hz and segmented into multiple files, each containing 4097 data points. The datasets from both sources were merged based on their respective categories, resulting in combined datasets of 200, 352, and 106 files for the three classes, respectively. These merged datasets were then used for augment.

### B. Data Augment

Acquiring a substantial amount of real data for seizure periods is challenging due to their short and irregular durations, privacy concerns related to EEG data, and the complexities of labeling. In order to solve the problem of small sample size and unbalanced categories in real data acquisition, we propose a method that mixes two data augmentation strategies. Our hybrid data augmentation strategy includes small window segmentation and SMOTE, which can effectively solve the problems of small data samples and unbalanced categories, respectively. Small window segmentation.

#### 1) Small Window Segmentation

The Small Window Segmentation algorithm divides lengthy data into multiple shorter segments by selecting an appropriate window size and sliding step. Assuming the length of the long data is $l$, the window size is $w$, and the sliding step is $s$, the number of short data segments (*num*) can be calculated using the following equation:

\begin{equation*} {num}=\frac {(l-w)}{s}+1 \tag{1}\end{equation*}

The integer part of num represents the actual number of generated data segments. The window size directly influences the size of the generated data; with a fixed network architecture, a larger window size yields larger data segments, which, after passing through the Bi-LSTM layer, could lead to an excessive number of connections to the fully connected layer, thus increasing the network parameters. However, a window size that is too small may fail to capture the intrinsic meaning of the data. The step size, on the other hand, determines the quantity of generated data. A smaller step size results in higher overlap between segments, potentially affecting data quality. Conversely, a larger step size generates fewer but potentially higher-quality instances, which is beneficial for training. Therefore, choosing the optimal window and step size is vital for ensuring data quality and effective training.

#### 2) SMOTE

Addressing the problem of epileptic EEG data classification using neural networks, it is crucial to ensure the model can fully learn the unique features of each category. This requires an equal amount of data from each category for model training. If data from a particular class is scarce, the model may not fully learn its features, leading to classification errors. However, it would not have a significant impact on overall classification accuracy due to the relatively small amount of data in this category. This can result in the model’s learned features being skewed towards the other classes, becoming overly specific and lacking generalization. To circumvent this issue, the SMOTE algorithm is employed to expand the epileptic EEG data of minority classes.

The algorithm generates new samples using the K-nearest neighbors algorithm and linear interpolation. The process involves selecting a reference sample $x_{i}$ from the minority class samples. Next, an auxiliary sample $x_{j}$ is randomly selected from the $k$ nearest neighbors of $x_{i}$ within the same category. This $k$ is the SMOTE upsampling ratio, which is the ratio of the number of samples in the majority category to the number in the minority category. If $k$ is not an integer, its fractional part is converted to a probability value for selecting neighboring samples. Linear interpolation is then performed between the reference sample $x_{i}$ and each auxiliary sample $x_{j}$, as defined in Equation (2).

\begin{equation*} x_{n e w}=x_{i}+\lambda \left ({x_{j}-x_{i}}\right) \tag{2}\end{equation*}

where $x_{new}$ is the newly generated sample with the same label as the reference sample, and $\lambda $ is a random number between [0, 1].

### C. PCNN-BiLSTM

As computational power escalates, neural network architectures grow increasingly intricate. While accuracy improves, deploying these networks for long-term epilepsy detection becomes a formidable challenge [31]. To facilitate the clinical application of deep learning techniques, the adoption of lightweight neural network architectures is advantageous. The proposed framework in this study encompasses two core modules: the PCNN and the Bidirectional Long Short-Term Memory (BiLSTM). These modules, leveraging their distinct computational approaches, collaboratively extract multi-dimensional features from raw EEG signals.

#### 1) PCNN

CNNs are a cornerstone of deep learning methodologies, prized for their robust representational learning capabilities. CNNs employ convolutional kernels to convolve input signals, generating multi-dimensional output features. Through a series of convolutions, pooling, and activation operations, these networks efficiently learn the most pertinent features. Shared kernel features in CNNs significantly reduce learning parameters compared to traditional fully connected networks. However, recent CNN structures have grown increasingly complex, encompassing more layers and, consequently, a larger number of parameters. Such structures, when applied to data-scarce tasks such as epileptic EEG recognition, tend to overfit.

To mitigate this issue, Ullah et al. [32] proposed the pyramid P-1D-CNN module. We use this module as part of our network architecture. Unlike conventional CNNs that employ pooling layers for size reduction, this model utilizes larger convolutional step sizes to extract more meaningful and distinctive features. By integrating numerous convolutional kernels at the shallow layer and fewer at the deep layer, the PCNN model significantly reduces the number of trainable parameters. This architectural adaptation not only simplifies the model’s complexity but also lessens the risk of overfitting. The entire structure comprises three consecutive convolutional blocks of decreasing sizes. Each block includes a convolutional layer (Conv), a batch normalization layer (BN), and a non-linear activation layer (ReLU), aiming to achieve optimal detection accuracy with minimal complexity.

#### 2) Bi-LSTM

EEG signals are temporal in nature, with potential interrelationships among the signals. To decipher this latent information, we employ LSTM, a type of RNN algorithm, renowned for modeling sequential data. Unlike standard RNNs that relay only a hidden state at each time step, LSTM tackles the issue of learning long-term dependencies by incorporating a cell state—essentially a long-term memory component that preserves vital information from preceding moments.

To counteract the problem of vanishing gradients during backpropagation and to ensure sustained learning, LSTM utilizes a dual memory system—comprising a hidden state and a cell state—those aids in retaining and propagating relevant information. The architecture of LSTM, illustrated in Figure 3, accepts three inputs for each LSTM block: the current network input value ( $X_{t}$), the previous LSTM hidden state ( $h_{t-1}$), and the previous LSTM cell state ( $c_{t-1}$). LSTM also generates two outputs: the updated hidden state ( $h_{t}$) and the updated cell state ( $c_{t}$).

LSTM’s operation is governed by three crucial parameters: the forget gate ( $f_{t}$), the input gate ( $i_{t}$), and the output gate ( $o_{t}$). These parameters modulate the behavior of the cell state ( $c_{t}$). Specifically, the forget gate determines the amount of information to be discarded from the cell state. Conversely, the input gate dictates the volume of new information to be incorporated into the cell state and subsequently outputs this updated cell state. Lastly, the output gate is responsible for generating the hidden state. The specific calculations for these gates are as follows:

\begin{align*} f_{t}&=\sigma \left ({W_{f}\left [{h_{t-1}, X_{t}}\right]+b_{f}}\right) \tag{3}\\ i_{t}&=\sigma \left ({W_{i}\left [{h_{t-1}, X_{t}}\right]+b_{i}}\right) \tag{4}\\ o_{t}&=\sigma \left ({W_{o}\left [{h_{t-1}, X_{t}}\right]+b_{o}}\right) \tag{5}\\ c_{t}^{\prime }&=tanh \left ({W_{c}\left [{h_{t-1}, X_{t}}\right]+b_{c}}\right) \tag{6}\\ c_{t}&=f_{t} * c_{t-1}+i_{t} * c_{t}^{\prime } \tag{7}\\ h_{t}&=o_{t} * tanh \left ({c_{t}}\right) \tag{8}\end{align*}

where $W_{f}$, ${b} _{f}$, $W_{i}$, ${b} _{i}$, $W_{o}$, ${b} _{o}$, $W_{c}$, ${b} _{c}$, are the training weights, $c_{t}^{\prime }$ is the new Input, and *tanh* and $\sigma $ *(sigmoid)* are the activation functions.

The Bidirectional-Long Short-Term Memory (Bi-LSTM) architecture is characterized by the amalgamation of two distinct LSTM networks. This arrangement enables parallel processing of inputs in both forward and backward sequences, facilitating comprehensive feature extraction. By merging the outputs of these two LSTM networks, a final output is generated, yielding a more comprehensive representation of the input data.

With this methodology, each data instance is analyzed from dual perspectives—considering not just preceding information but also subsequent information. Consequently, the Bi-LSTM model captures a broader perspective of the input sequence, enabling the extraction of features with a larger global context compared to conventional LSTM models.

### D. Evaluation Metrics

In the realm of multi-classification problems, algorithm performance assessment often employs a confusion matrix. This matrix forms the foundation for four fundamental evaluation metrics: Accuracy (Acc), Sensitivity (Sens), Specificity (Spec), and Precision (Prec).The calculated formulae are as follows:

\begin{align*} A c c&=\frac {T P+T N}{T P+T N+F P+F N} \tag{9}\\ {Sens}&=\frac {T P}{T P+F N} \tag{10}\\ {Spec}&=\frac {T N}{F P+T N} \tag{11}\\ {Prec}&=\frac {T P}{T P+F P} \tag{12}\end{align*}

These metrics elucidate the results of the algorithm’s classification. Within the confusion matrix framework, several pivotal terms are used to calculate these metrics. True Positive (TP) signifies situations where the sample is positive, and the algorithm correctly identifies it as such. True Negative (TN) pertains to instances where the sample is negative, and the algorithm correctly classifies it as negative. False Positive (FP) embodies scenarios where the sample is negative, but the algorithm erroneously classifies it as positive. Lastly, False Negative (FN) includes cases where the sample is positive, but the algorithm incorrectly classifies it as negative.

## IV. Experimental Settings

### A. Experimental Environment

The experimental setup for this study leveraged a 64-bit Windows 10 operating system. The computational workload was handled by an Intel(R) Core(TM) i5-7200U CPU. The programming language of choice was Python 3.9, and TensorFlow 2.10.0 was utilized as the deep learning framework.

### B. Experimental Procedure

The experimental procedure in this study is bifurcated into two core segments: data augmentation, feature extraction and classification. To enhance the network’s generalization capability, a 10-fold cross-validation method was employed for training. The dataset was partitioned into a training set and a test set in a 9:1 ratio, culminating in 10 subsets. Sequentially, 9 subsets were used for training and 1 subset was reserved for testing, with each subset iteratively serving as the test data.

#### 1) Data Augmentation

The data augmentation segment includes small window segmentation and the SMOTE. During the small window segmentation phase, choosing suitable window and step sizes is vital for producing high-quality data. To ascertain the optimal segmentation size, several experiments were performed to compare the classification performance of different methods with window sizes of 1024, 512, 256, and 128, and step sizes of 64 and 32, respectively (refer to Table 1 for specific experimental parameters).

**TABLE 1:** Parameters of the Models

Model	Window	Stride	SMOTE
Model 1	128	64	Yes
Model 2	256	64	Yes
Model 3	512	64	Yes
Model 4	1024	64	Yes
Model 5	128	32	Yes
Model 6	256	32	Yes
Model 7	512	32	Yes
Model 8	1024	32	Yes
Model 9	128	64	No
Model 10	256	64	No
Model 11	512	64	No
Model 12	1024	64	No
Model 13	128	32	No
Model 14	256	32	No
Model 15	512	32	No
Model 16	1024	32	No

The SMOTE algorithm tackles the issue of imbalanced sample distribution across categories. The mixed dataset used in this study consists of three categories: normal, interictal, and ictal, with 200, 352, and 106 data samples in each category, respectively. To assess the effectiveness of the SMOTE algorithm, two experiments were conducted using the same model—one with the SMOTE algorithm and another without any data balancing process (refer to Table 1 for specific experimental parameters).

#### 2) Feature Extraction and Classification

The feature extraction and classification phase is executed using a PCNN-BiLSTM hybrid network. Initially, the data undergoes normalization to improve convergence speed and accuracy, and to prevent gradient explosion. Following this, the normalized data is fed into the neural network. The network’s weight is optimized by minimizing parameters in each layer, ensuring a lightweight structure while preserving prediction efficacy. The proposed PCNN architecture comprises three convolutional blocks, with kernel sizes set at 5, 3, and 3, and step sizes at 3, 2, and 2, respectively. For detailed specifications of each network layer, refer to Table 2.

**TABLE 2:** Detailed Parameters of the Layers

Layer	Type	Kernel number, size, stride	Dropout	Activate function
Input	$256\ast 1$	–	–	–
Conv-1	Conv	24, $1\ast 5$,3	–	ReLU
Conv-2	Conv	16, $1\ast 3$,2		ReLU
Conv-3	Conv	8, $1\ast 3$,2	–	ReLU
Bi-LSTM	Bi-LSTM	$20\ast 8$	–	–
Dropout	Dropout-1	–	0.1	–
FC	FC-1	20	–	ReLU
Dropout	Dropout-2	–	0.5	–
FC	FC-2	3	–	SoftMax

For training the entire network, a cross-entropy loss function and an Adam optimizer are utilized. The hyperparameters of the Adam optimizer are set as follows: learning rate (0.00002), beta1 (0.9), beta2 (0.999), epsilon (0.00000001), decay (0.0), and amsgrad (False). The training process spans 1000 epochs as determined for this experiment.

## V. Results

Due to the limited number of training samples, the data used in our experiment is augmented using small window segmentation and SMOTE algorithms. The small window segmentation experiments involved eight models (Model 1-Model 8), using four different window sizes (128, 256, 512, and 1024) and two sliding step sizes (32 and 64). All other parameters remained constant for comparative analysis. The results, visualized in Fig 6, were evaluated accordingly.

**FIGURE 4.**
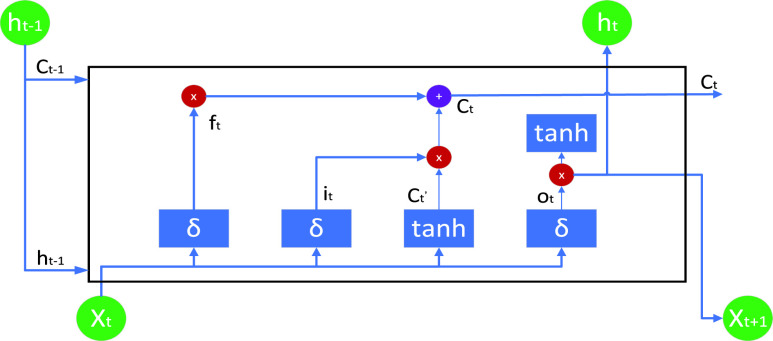
LSTM architecture.

**FIGURE 5.**
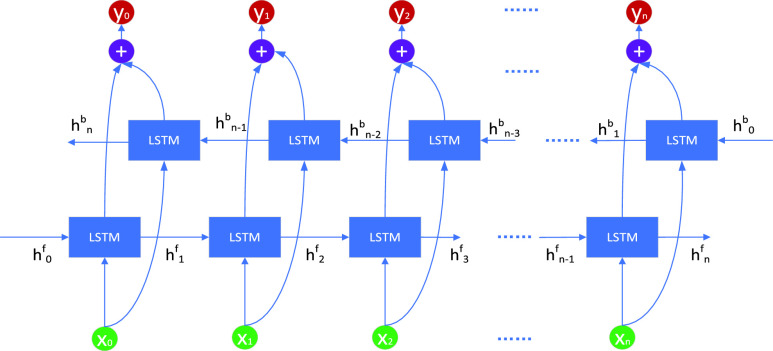
Bi-LSTM.

**FIGURE 6.**
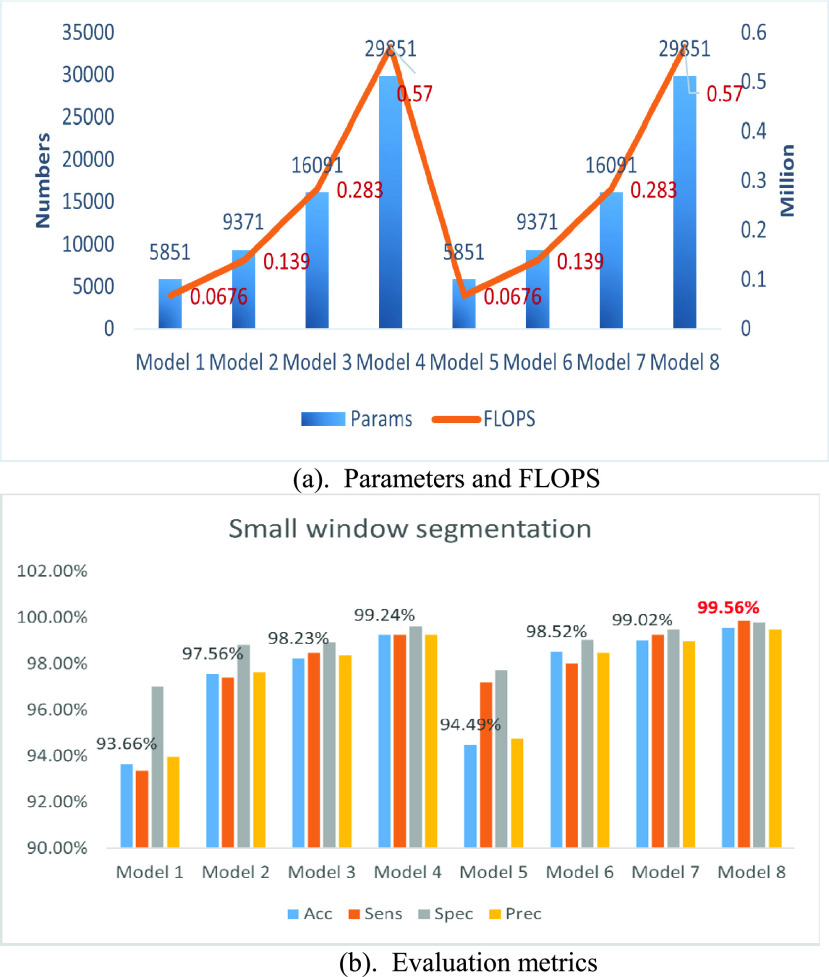
(a) Parameters and FLOPS. (b) Evaluation metrics.

Fig 6(a) illustrates the relationship between the number of model parameters and the corresponding FLOPS (Floating Point Operations Per Second). FLOPS is a metric for measuring computational load in a neural network, representing the number of floating point operations per second in millions. Higher FLOPS values signify increased computational operations, implying greater computational demands and higher hardware requirements.

The results indicate that the number of model parameters is solely influenced by the window size, showing an almost exponential increase as the window size enlarges exponentially. This pattern is similarly observed in the FLOPS value.

Fig 6(b) demonstrates that, for a given stride size, the model’s accuracy improves with larger window sizes, especially when transitioning from a window size of 128 to 256, where significant performance improvements are observed. However, further increases in window size result in marginal improvements of about 1%. Additionally, the model’s accuracy improves by roughly 1% for each metric when using smaller stride sizes at a fixed window size. Notably, the network achieves an accuracy of 99.56% (Model 8) without considering computational complexity, underscoring the effectiveness of the proposed network structure.

In the SMOTE algorithm experiments, the SMOTE algorithm was used for the first 8 models (Models 1-8), while the last 8 models (Models 9-16) acted as a control group without the SMOTE algorithm. This control group was used to validate the algorithm’s effectiveness.

**FIGURE 7.**
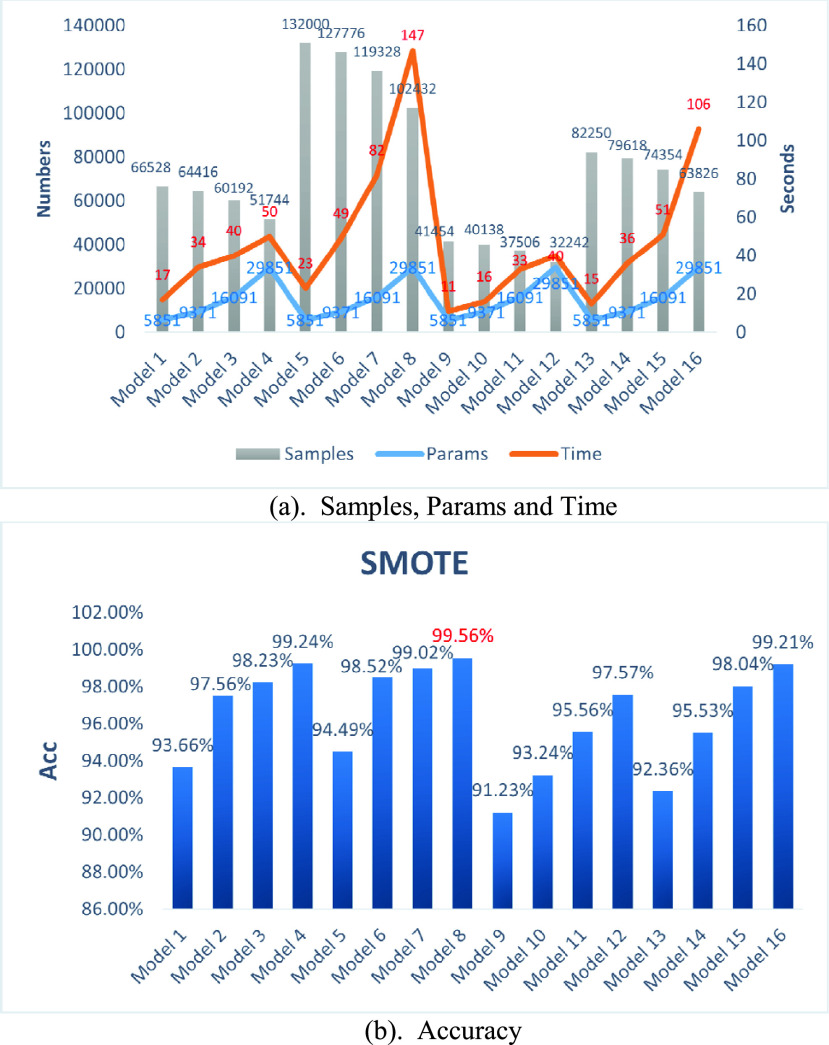
(a) Samples, Params and Time. (b) Accuracy.

Fig 7(a) displays the number of samples used for training each model, the model parameters, and the time required for training per epoch. Analysis of the test results shows that, after applying the SMOTE algorithm, the total number of samples in Models 1 to 8 increased by 60.48%, resulting in an equal number of samples across all classes.

The training time was influenced by both the number of network parameters and the number of samples, with the former having a more significant impact, while the latter had a relatively minor effect. Therefore, when choosing a model, it is recommended to select one with fewer parameters and a larger sample size. It should be noted that while the training time is significantly affected by the experimental environment (CPU load status), this experiment aimed to maintain a consistent experimental environment as much as possible. As a result, the collected data somewhat reflects the model’s performance.

In Fig 7(b), among the twelve models with window sizes of 256, 512, and 1024, significant improvements were observed in Models 2 to 8 (except Model 5) with the SMOTE algorithm compared to Models 10 to 16 (except Model 13) without it. Specifically, for the 4 models with a window size of 256, there were substantial improvements in the Acc indicator of around 3% to 4%, which is considered a significant advancement.

However, in the 4 models with a window size of 128, Model 9 and Model 13 without the SMOTE algorithm showed slightly better indicators than Model 1 and Model 5 with the SMOTE algorithm. This can be attributed to the poor quality of the data after small window segmentation, which is unsuitable for network training. Furthermore, the use of the SMOTE algorithm further exacerbates the data quality issue, thereby reducing the effectiveness of the network.

To build a lightweight framework, the preferred approach is to adopt a data processing scheme with fewer trainable parameters and reduced training time, while still achieving satisfactory accuracy. After considering the aforementioned experimental results, a processing scheme using a window size of 256, a sliding step size of 32, and incorporating the SMOTE algorithm (Model 6) was identified as the optimal solution. Table 3 presents the detailed training results for each metric of Model 2.

**TABLE 3:** Fold Training Metrics

	Acc	Sens	Spec	Prec
Fold 1	98.13%	98.08%	99.66%	98.32%
Fold 2	98.97%	97.94%	99.53%	98.06%
Fold 3	98.56%	98.40%	99.82%	98.62%
Fold 4	98.02%	97.03%	98.87%	98.12%
Fold 5	98.87%	98.55%	99.54%	98.69%
Fold 6	98.54%	98.36%	99.57%	98.56%
Fold 7	98.48%	97.12%	98.35%	98.41%
Fold 8	98.13%	98.49%	98.91%	98.15%
Fold 9	98.65%	97.20%	99.54%	98.67%
Fold 10	98.87%	98.71%	99.68%	98.79%
Average	98.52%	97.99%	99.35%	98.44%

**FIGURE 8.**
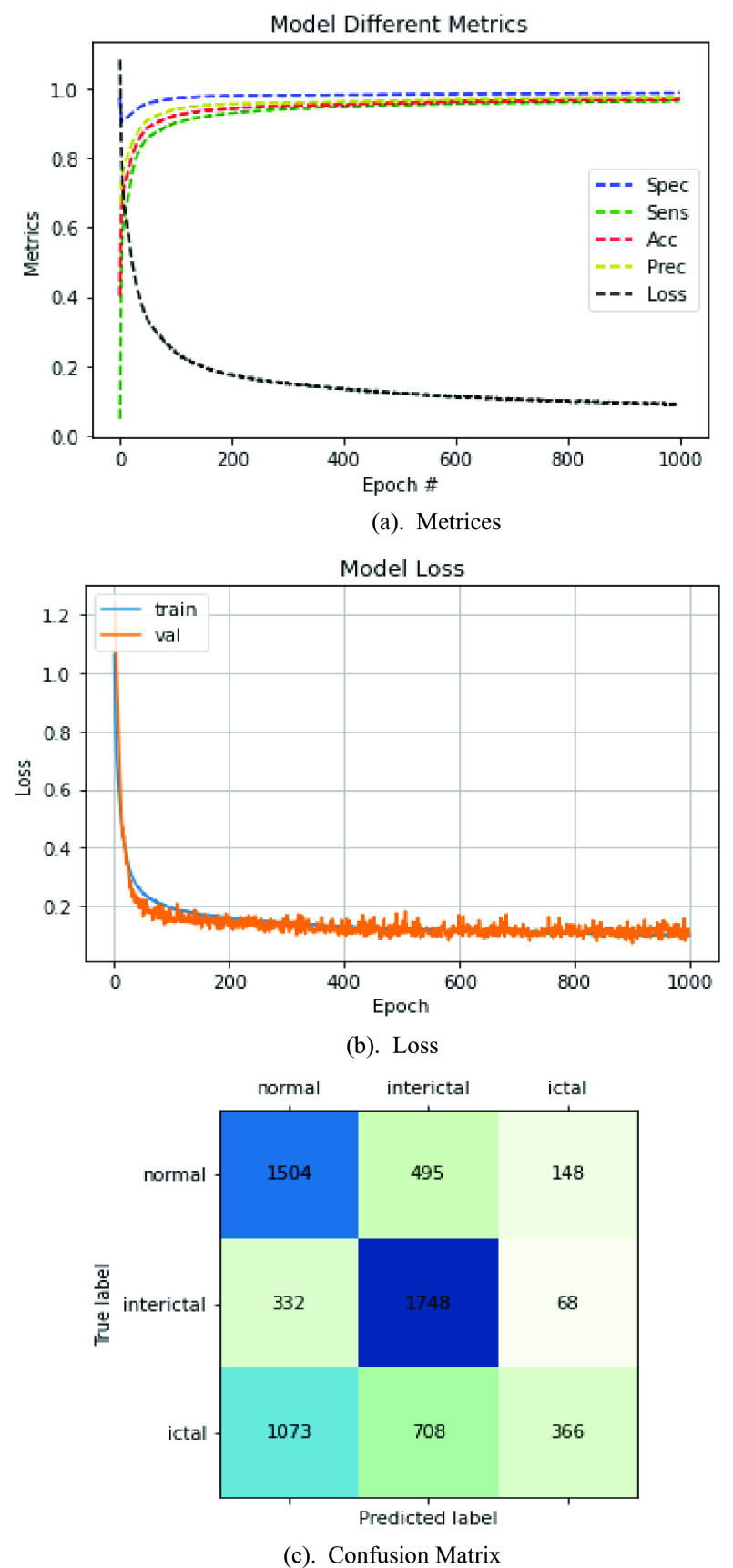
Metrices. (b) Loss. (c) Confusion Matrix.

The ten-fold cross-validation resulted in an average Acc of 98.52%, Sens of 97.99%, Spec of 99.35%, and Prec of 98.44% for the comprehensive model, all of which indicate favorable results. Figures 8(a), 8(b), and 8(c) depict the fluctuation curves and the categorized confusion matrices for each metric, showing a smooth transition for all the metrics and gradual convergence of the model as the epoch increases.

## VI. Discussion

Table 4 presents a comparison of studies that utilize the Bonn University and CHB-MIT datasets for epileptic EEG classification, thereby underscoring the sophistication and effectiveness of the framework proposed in this research.

**TABLE 4:** Comparison Between Studies

Author	Model	Dataset	Params	Acc
Rajendra et al. [18](2018)	1DCNN	Bonn	96190	88.7
David et al. [33]	LSTM	Bonn	116033	91.25
Vidyaratne et al. [19] (2022)	DCRNN	CHB-MIT	N.A	91.3
Shankar et al. [34](2021)	2DCNN	Bonn	846290	93
Ramy et al. [35] (2019)	LSTM	Bonn	8218763	95.2
Pritam et al. [36] (2021)	HVD^a^-LSTM	Bonn	654003	96
Thuwajit et al. [37] (2022)	EEGWaveNet	Bonn/CHB-MIT	44776	95.27/96.17
Salafian et al. [25](2023)	MICAL	CHB-MIT	N.A	95.39
Ulas et al. [38](2019)	1DCNN-BiLSTM	Bonn	13198211	97
Qiu et al. [21] (2023)	LightSerizueNet	CHB-MIT	1983000	97.09
Shyu et al. [39] (2023)	LBPMAD^b^	Bonn	N.A	97.13
Shen et al [20] (2023)	Q-wavelet tansform+CNN	CHB-MIT	N.A	97.57
Tang et al. [22] (2023)	ESN+FDEF^c^	Bonn/CHB-MIT	N.A	98.1/96.14
Shyu et al. [28](2023)	Inception+Residual	CHB-MIT	28590	98.34
This work	PCNN-BiLSTM	Bonn&CHB-MIT	9371	98.52

^a^ Hilbert vibration decomposition

^b^ Local Binary Pattern Mean Absolute Deviation

^c^ Echo State Network+Feature Distribution Evaluation Function

Among the comparison methods discussed above, the approaches proposed in [21] and [38] both achieve an accuracy of approximately 97%, but their network parameters are extensive, hundreds or even over a thousand times that of the methods proposed in this study. This makes them more challenging to train and places higher demands on hardware equipment. References [22] and [39] employ traditional machine learning methods, and [20] uses a combination of deep learning and machine learning methods. Both of these methods also yield good recognition results, but their principles are complex and require manual extraction of the right features. Reference [28] employs deep learning to achieve a high accuracy rate with fewer overall parameters. In the PCNN-BiLSTM framework proposed in this study, the parameters to be trained are much less than these methods, and at the same time, our method achieved 98.52% accuracy. Moreover, none of the aforementioned methods adopt data augmentation algorithms, and the trained networks lack generalization.

From a comprehensive perspective, the data augmentation and lightweight neural network-based approach proposed in this study has a competitive recognition accuracy and low parametric count. By using a mixed dataset for training and incorporating the data augmentation algorithm, the model demonstrates robust generalization. Furthermore, the entire network architecture is compact, with only 9371 parameters to be trained, a model size of merely 186kb, and a FIOPS of 0.139M. This minimal resource requirement makes it well-suited for applications in clinical medical devices and wearables, thereby offering a significant advantage over recent research advances.

However, there are limitations to this study that warrant consideration. First, although data augmentation techniques are used to expand the dataset, the quality of the generated data still falls short of real-world data. Second, validation on hardware devices has not been conducted. Lastly, the impact of various factors in real life on model recognition performance needs to be more thoroughly considered. These factors include device aging, discomfort from prolonged usage, and emotional fluctuations due to extended wearing of the device, all of which may lead to a decline in signal quality.

## VII. Conclusion

In this study, a novel lightweight PCNN-BiLSTM hybrid network is presented, coupled with a data augmentation algorithm for the detection of epileptic EEG. The employed methodology involves segmenting the original data using a small window technique to increase the sample size, followed by the application of the SMOTE algorithm to balance the class distribution by expanding minority class samples. The processed data is then introduced into a neural network for feature extraction and classification via the PCNN-Bi-LSTM architecture. Rigorous validation on the epileptic EEG datasets from Bonn University and CHB-MIT yielded impressive classification accuracies of 98.52%, demonstrating a high level of competitiveness in the field with only 9371 trainable parameters.

Future work is warranted to further enhance the performance and applicability of the proposed method. This includes training the network with lower-quality data, which more accurately represents real-world conditions, and revising the network code to facilitate deployment and further validation on hardware devices. These improvements will augment the model’s compatibility with practical application scenarios, thereby enhancing its robustness and reliability to cater to a wide array of real-world requirements.
